# [Retracted] miR‑17‑5p promotes the growth of osteosarcoma in a BRCC2‑dependent mechanism

**DOI:** 10.3892/or.2024.8710

**Published:** 2024-01-29

**Authors:** Wei Wang, Liang Zhang, Ke Zheng, Xiaojing Zhang

Oncol Rep 35: 1473–1482, 2016; DOI: 10.3892/or.2016.4542

Following the publication of this paper, it was drawn to the Editor's attention by a concerned reader that the western blotting data shown in Fig. 7 on p. 1480 were strikingly similar to data that had already been published in another article written by different authors at different research institutes, which has subsequently been retracted [Fan C, Wang Y, Liu Z, Sun Y, Wang X, Wei G and Wei J: Metformin exerts anticancer effects through the inhibition of the Sonic hedgehog signaling pathway in breast cancer. Int J Mol Med 36: 204-214, 2015].

Owing to the fact that the contentious data in the above article were already under consideration for publication prior to its submission to *Oncology Reports*, the Editor has decided that this paper should be retracted from the Journal. The authors were asked for an explanation to account for these concerns, but the Editorial Office did not receive a reply. The Editor apologizes to the readership for any inconvenience caused.

